# Nitrogen Preferences during Alcoholic Fermentation of Different Non-*Saccharomyces* Yeasts of Oenological Interest

**DOI:** 10.3390/microorganisms8020157

**Published:** 2020-01-22

**Authors:** Helena Roca-Mesa, Sonia Sendra, Albert Mas, Gemma Beltran, María-Jesús Torija

**Affiliations:** Departament de Bioquímica i Biotecnologia, Grup de Biotecnologia Enològica, Facultat d’Enologia, Universitat Rovira i Virgili, c/ Marcel·lí Domingo, 1. 43007 Tarragona, Spain

**Keywords:** wine fermentation, *Torulaspora delbrueckii*, *Lachancea thermotolerans*, *Starmerella bacillaris*, *Hanseniaspora uvarum*, *Metschnikowia pulcherrima*, amino acids, ammonium

## Abstract

Non-*Saccharomyces* yeasts have long been considered spoilage microorganisms. Currently, oenological interest in those species is increasing, mostly due to their positive contribution to wine quality. In this work, the fermentative capacity and nitrogen consumption of several non-*Saccharomyces* wine yeast (*Torulaspora delbrueckii*, *Lachancea thermotolerans*, *Starmerella bacillaris*, *Hanseniaspora uvarum*, and *Metschnikowia pulcherrima*) were analyzed. For this purpose, synthetic must with three different nitrogen compositions was used: a mixture of amino acids and ammonium, only organic or inorganic nitrogen. The fermentation kinetics, nitrogen consumption, and yeast growth were measured over time. Our results showed that the good fermentative strains, *T. delbrueckii* and *L. thermotolerans*, had high similarities with *Saccharomyces cerevisiae* in terms of growth, fermentation profile, and nitrogen assimilation preferences, although *L. thermotolerans* presented an impaired behavior when only amino acids or ammonia were used, being strain-specific. *M. pulcherrima* was the non-*Saccharomyces* strain least affected by the nitrogen composition of the medium. The other two poor fermentative strains, *H. uvarum* and *S. bacillaris*, behaved similarly regarding amino acid uptake, which occurred earlier than that of the good fermentative species in the absence of ammonia. The results obtained in single non-*Saccharomyces* fermentations highlighted the importance of controlling nitrogen requirements of the wine yeasts, mainly in sequential fermentations, in order to manage a proper nitrogen supplementation, when needed.

## 1. Introduction

Wine is a common product that has been consumed since antiquity and is the result of several biochemical reactions produced principally by yeasts. During alcoholic fermentation, yeasts transform sugars into ethanol and carbon dioxide. Additionally, several metabolites are involved in this process and are present in the final product, contributing to the final quality and complexity of the wine [1]. Although the main yeast responsible for wine fermentation is *Saccharomyces cerevisiae*, there are also non-*Saccharomyces* yeast species involved in the process. Those non-*Saccharomyces* species can be found on the grape surface and, for many years, have been considered spoilage microorganisms. Currently, it has been demonstrated that some non-*Saccharomyces* yeasts can positively contribute to the organoleptic profile of wines, producing volatile compounds not produced by *Saccharomyces* strains, consequently providing different characteristics to the final product [2,3,4,5]. Therefore, the use of different non-*Saccharomyces* species in mixed fermentation with *S. cerevisiae* is a good alternative to improve certain wine characteristics. For example, it has been shown that *Torulaspora delbrueckii* enhances the complexity and fruity notes of wines [6], *Hanseniaspora vineae* enriches wines with fruity and flowery aromas [7], *Lachancea thermotolerans* increases the total acidity [8], and *Metschnikowia pulcherrima* reduces the ethanol levels and enhances varietal aromas [9,10]. However, to optimize the use of non-*Saccharomyces* yeasts in sequential and co-inoculated fermentations with *Saccharomyces* spp., it is necessary to better understand their metabolism and nutrient requirements. During a sequential inoculation, the initial consumption of nutrients by non-*Saccharomyces* yeasts could affect the growth and survival of *Saccharomyces* yeasts, inoculated later [2,11,12].

Nitrogen compounds are the nutrients mostly assimilated by yeasts, after carbon compounds, during alcoholic fermentation. They are involved in the metabolism and growth of yeasts, affecting the correct evolution of the fermentation [1,13], and the production of volatile compounds [14,15,16]. In grape musts, nitrogen composition can be highly variable, both in concentration and in the types of nitrogen compounds present [17]. Furthermore, only some of these nitrogen compounds, known as yeast assimilable nitrogen (YAN), are metabolized by yeast. In *S. cerevisiae*, YAN sources include amino acids and ammonia [18]. YAN can be classified into two categories: preferred and non-preferred nitrogen sources [19]. The preferred nitrogen sources, such as ammonia, glutamine, and asparagine, promote yeast growth. Instead, non-preferred sources, such as proline and urea, result in low growth when they are the only nitrogen sources. Nitrogen deficiencies are the common causes of sluggish or stuck fermentation [13,20,21]. For this reason, in some cases, natural musts are supplied with external nutrients, typically ammonium salts, to avoid fermentative problems [22]. However, Gutiérrez et al. [23] showed that different commercial *S. cerevisiae* strains had different nitrogen needs and proposed the use of supplements containing organic nitrogen sources.

While nitrogen consumption and preferences are well researched in *S. cerevisiae* [17,23,24,25,26,27,28,29,30,31], nutrient uptake by non-*Saccharomyces* yeasts has not been extensively studied. Available studies have analyzed the preferential nitrogen sources of some non-*Saccharomyces* yeasts, focusing on their capacity to assimilate different nitrogen compounds, their rate of consumption, or their influence in aroma production [2,19,32,33,34,35,36,37,38]. The firsts studies reported that the nitrogen sources directly affect the fermentation performance and yeast growth in a species-specific manner [32,39]. Later, some studies have revealed that non-*Saccharomyces* yeasts have specific profiles for amino acid consumption, concluding that the different nitrogen composition of the media strongly influences the assimilation order of each compound, both in natural [19] and synthetic must [12,35,37].

Different studies have analyzed the possible competition for nutrients between *Saccharomyces* and non-*Saccharomyces* yeasts [11,12,40,41,42]. Rollero et al. [12] observed different consumption profiles of *S. cerevisiae* depending on the non-*Saccharomyces* yeasts used in the sequential culture, suggesting that this behavior could be explained by the competition for nutrients. Moreover, recent studies have demonstrated that under conditions of co-cultivation with some non-*Saccharomyces* species, *S. cerevisiae* partially relieves the nitrogen and glucose catabolite repression, in order to increase the flux of nutrients and reduce their availability for other yeast species [12,40]. Indeed, the presence of other *Saccharomyces* species, such as *Saccharomyces kudriavzevii*, can also produce metabolic stimulation in *S. cerevisiae* [41].

Thus, the increasing popularity of non-*Saccharomyces* yeasts in winemaking makes it necessary to know more about them, especially about their nitrogen preferences. With this knowledge, winemakers can improve non-*Saccharomyces* implementation and avoid problems during alcoholic fermentation. Some of these non-*Saccharomyces* species, such as *Torulaspora delbrueckii*, *Lachancea thermotolerans*, and *Metschnikowia pulcherrima*, are already on the market as active dry yeast [33], but other species with great potential in the enological industry are still being evaluated.

The aim of this study was to analyze the nitrogen consumption and fermentative kinetics of commercial and non-commercial non-*Saccharomyces* yeasts. We performed fermentations on a laboratory scale using synthetic must with different nitrogen sources: only amino acids, only ammonium, or a combination of amino acids and ammonium. The fermentation kinetics, yeast growth, and nitrogen consumption were monitored throughout the fermentation, comparing the results of these non-*Saccharomyces* with those of a commercial *S. cerevisiae* strain used as a control.

## 2. Materials and Methods

### 2.1. Yeast Strains

Six yeast species were used in this study. The non-*Saccharomyces* strains were *Torulaspora delbrueckii* Viniferm NS-TD (Agrovin S.A., Spain) (Td), *Lachancea thermotolerans* Lt2 provided by Agrovin S.A. (Lt), *Hanseniaspora uvarum* CECT 1444 (from Spanish Type Culture Collection) (Hu), and *Metschnikowia pulcherrima* CECT 13131 (Mp) and *Starmerella bacillaris* CECT 13129 (Sb) isolated from Priorat DOQ (Qualified Designation of Origin) (URV collection). *Saccharomyces cerevisiae* Viniferm Revelación (Agrovin S.A.) (Sc) was used as a control. To confirm the nitrogen preferences of *L. thermotolerans*, other strains were analyzed: LAKTIA (Lallemand S.L.) (LtK), Lt1 provided by Agrovin S.A. (Lt1) and *L. thermotolerans* ICVV1131 (Lt78), ICVV1132 (Lt79) and ICVV1133 (Lt80), from the ICVV collection (Logroño, Spain).

All strains were preserved in YPD liquid medium (2% (w/v) glucose, 2% (w/v) bacto peptone, and 1% (w/v) yeast extract; Cultimed, Barcelona, Spain) with 40% (v/v) glycerol, at –80 °C. Before their use, they were streaked on YPD agar plates (YPD liquid with 2% (w/v) agar). Isolated colonies from these pure cultures were grown in YPD at 28 °C and 120 rpm in an orbital shaker for 24 h and used as a preculture for inoculating the fermentations. The microscopic counting of the cells by a Neubauer chamber was used to calculate the cell concentration in the precultures.

### 2.2. Fermentation Conditions and Sampling

Single inoculum fermentations were performed in synthetic must, as described in Beltran et al. [43] (Table S1). The initial nitrogen content was 300 mg N/L in all cases (Table S2), but different nitrogen compositions were used: 1) the control condition with 40% ammonium and 60% amino acids (SM-Mix), 2) amino acids as a sole nitrogen source (SM-AA), and 3) only ammonium (SM-NH_4_^+^). Synthetic must was inoculated to a final concentration of 2 × 10^6^ cells/mL, for each yeast species: *S. cerevisiae* (Sc), *T. delbrueckii* (Td), *L. thermotolerans* (Lt), *H. uvarum* (Hu), *M. pulcherrima* (Mp), and *S. bacillaris* (Sb). Fermentations were performed in triplicate at 22 °C and 120 rpm in 250 mL borosilicate glass bottles containing 220 mL of medium and capped with closures that enabled carbon dioxide to escape and samples to be removed. Fermentations were conducted under semi-anaerobic conditions since some aeration was necessary for harvesting samples for subsequent analysis.

Fermentation kinetics were monitored by measuring the daily must density with an electronic densimeter (Densito 30PX Portable Density Meter (Mettler Toledo, España)), total yeast population by optical density at 600 nm (OD_600_), and viable yeast population by plating serial dilutions of samples on YPD and Wallerstein laboratory nutrient agar (WLN) medium (DifcoTM, Sparks, NV, USA). We selected the endpoint of fermentation when sugars were below 2 g/l, or in the case of stuck or sluggish fermentations, samples at 240 h were collected as end-point. For the analysis of the nitrogen compounds or organic metabolites, 1.5 mL of the supernatant was collected at 12, 24, 48, and 72 h and at the endpoint of fermentation (216 or 240 h) and stored at −20 °C, until analysis. Fermentations with different Lt strains were followed by lost weight in 40 mL of each medium.

### 2.3. Nitrogen Analysis

The nitrogen content was analyzed by HPLC (high-performance liquid chromatography), according to the method of Gómez-Alonso et al. [44]. The HPLC (Agilent 1100, Agilent Technologies, Germany) was equipped with a DAD ultraviolet detector and a fluorescence detector (Agilent Technologies, Germany), and separation was performed on a Hypersil ODS C18 column (Agilent Technologies, Germany) with a particle size of 5 μm (250 mm × 4.6 mm) and thermostated at 20 °C. The mobile phase (A) consisted of 2.05 g/L of sodium acetate anhydrous and 0.2 g/L of sodium azide with MilliQ water (Millipore Q-PODTM Advantage A10) adjusted to pH 5.8 with glacial acetic acid, and mobile phase (B) consisted of 80% (v/v) acetonitrile and 20% (v/v) methanol. Chromatograms were analyzed using Agilent ChemStation Plus software (Agilent Technologies, Germany). Amino acid and ammonium concentrations were transformed into yeast assimilable nitrogen (YAN, expressed as mg N/L) according to the nitrogen atoms of each amino acid. The ammonia concentration of SM-NH4^+^ was also analyzed with a multi analyzer Miura One (TDI, Barcelona, España), using an ammonia nitrogen enzymatic kit (TDI, Barcelona, España).

### 2.4. Metabolite Analysis

The concentration of organic metabolites (glucose, fructose, glycerol, ethanol, and acetic acid) was determined at 48 and 240 h (216 h in Sc), following the protocol described by Quirós et al. [45], using an Agilent 1100 HPLC (Agilent Technologies, Germany) equipped with a Hi-Plex H, 300 mm x 7.7 mm column inside a 1260 MCT ( Infinity II Multicolumn Thermostat) connected to both an MWC and an RID (G1365B multi-wavelength detector and 1260 Infinity II refractive index detector) (Agilent Technologies, Germany). The column was maintained at 60 °C, and 5 mM H_2_SO_4_ was used as the mobile phase at a flow rate of 0.6 mL/min. Previously, samples were filtered through 0.22 μm pore size filters (Dominique Dutscher, Brumath, France).

### 2.5. Statistical Analysis

Data are expressed as the mean and standard deviation of triplicates as data points. ANOVA and Tukey’s test analyses using XLSTAT 2019 software (Addinsoft, New York, NY, USA) were performed to determine significant differences between different nitrogen sources, media, and strains. The results were considered statistically significant at a *p*-value of less than 0.05. The GraphPad Prism 7 program (GraphPad Software, San Diego, CA, USA) was used for graphical data modeling.

## 3. Results

### 3.1. Fermentation Kinetics

**Figure 1:**
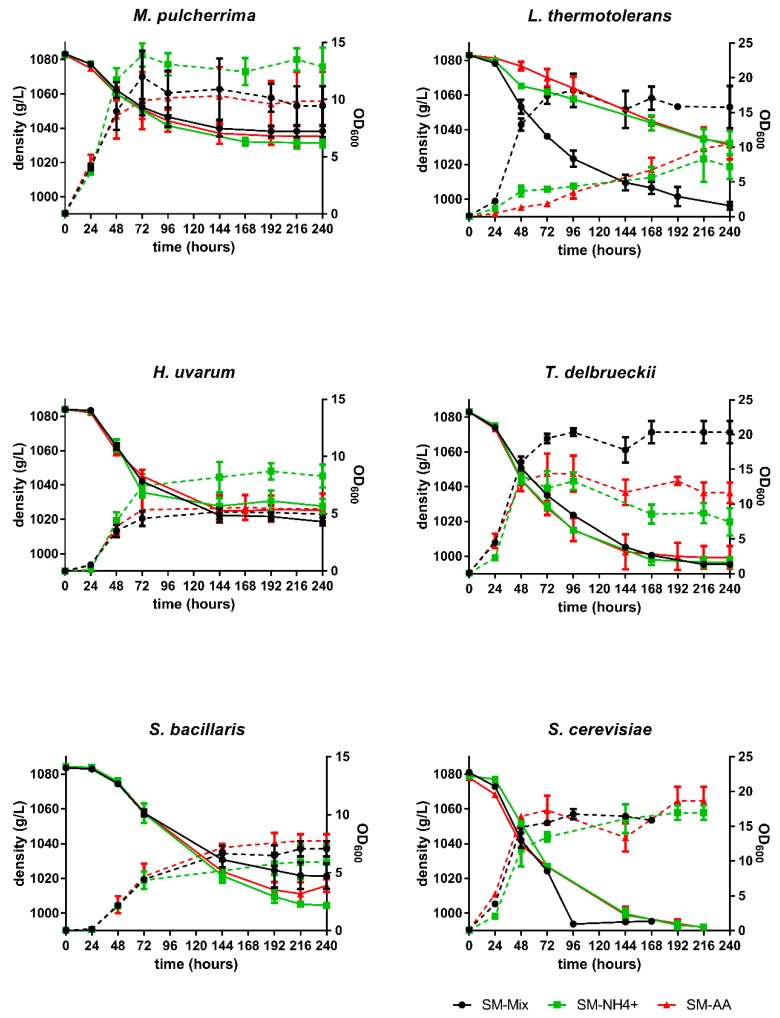
Fermentation kinetics and yeast growth of each species in synthetic must with different nitrogen sources: inorganic nitrogen only (SM-NH_4_+), organic nitrogen only (SM-AA), and a mixture of organic and inorganic nitrogen (SM-Mix). Solid lines refer to density and dotted lines refer to OD_600_. Error bars represent standard deviation.

Single fermentations with the five non-*Saccharomyces* species (*T. delbrueckii* (Td), *L. thermotolerans* (Lt), *H. uvarum* (Hu), *M. pulcherrima* (Mp), and *S. bacillaris* (Sb)), as well as a *S. cerevisiae* (Sc) strain as a control, were performed in synthetic must with different nitrogen sources: inorganic nitrogen only (SM-NH_4_^+^), organic nitrogen only (SM-AA), and a mixture of organic and inorganic nitrogen (SM-Mix) (Figure 1).

Among the non-*Saccharomyces* strains, only Td was able to finish the fermentation under all tested nitrogen conditions (10 days for all of them), although the best growth was obtained in SM-Mix. Lt growth and fermentation were impaired in SM-NH_4_^+^ and SM-AA. In these cases, the fermentations were sluggish and stuck at approximately 1040 g/L density, while the fermentation was complete in 10 days in the SM-Mix condition. For Hu and Mp, although cell growth was favored in SM-NH_4_^+^, the fermentations were stuck in all conditions at a similar density (between 1020 and 1040 g/L). Sb was not able to finish the fermentation under any of the nitrogen conditions, and the least amount of sugars were consumed in the SM-Mix fermentation. As expected, the fermentations with Sc were faster than those with the non-*Saccharomyces* strains finishing in 7 days for SM-Mix and in 9 days for SM-AA and SM-NH_4_^+^ (Figure 1).

We observed significant differences in growth between media in Lt, Td, and Hu (Table 1), being SM-Mix the best medium for Td and Lt growth, and SM-NH_4_^+^ the best for Hu. Moreover, Td was the non-*Saccharomyces* strain that grew best in all media, and Hu and Sb the ones with the lowest growth.

The species most affected by the nitrogen composition in the medium was Lt, as it presented good growth in the SM-Mix but poor growth when only ammonia or only amino acids were present, which was related to the sluggish fermentation observed under these conditions (Figure 1, Table 2).

**Figure 2:**
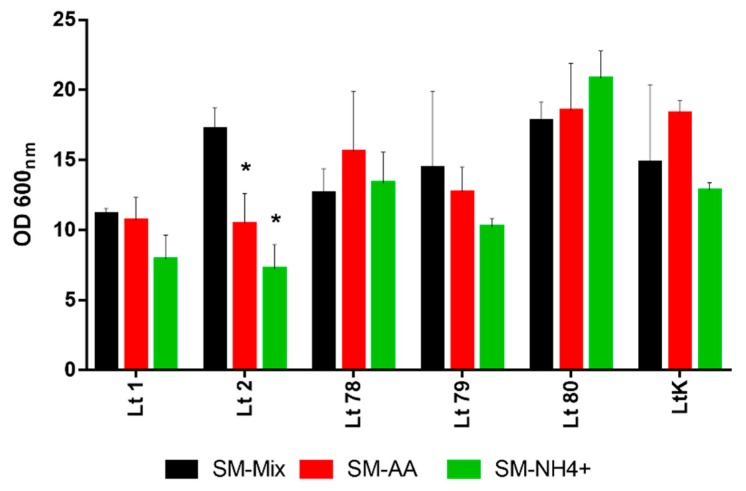
Maximal growth (expressed as OD_600_) of different *L. thermotolerans* strains at the end of fermentation in synthetic must with different nitrogen sources. Error bars represent standard deviation. * indicates statistically significant differences between media (*p* < 0.05).

To verify this profile, additional strains of *L. thermotolerans* were analyzed (Figure 2). Our results showed that nitrogen preferences were strain-dependent, as some of the strains exhibited better growth in SM-Mix (Lt2 and Lt79) and others in the medium with only amino acids (SM-AA, Lt78, and LtK). In general, ammonium was the nitrogen source that resulted in a low growth, except for the strain Lt80, which also fermented faster in this medium (Figure S1).

### 3.2. Nitrogen Consumption and Preferences

**Figure 3:**
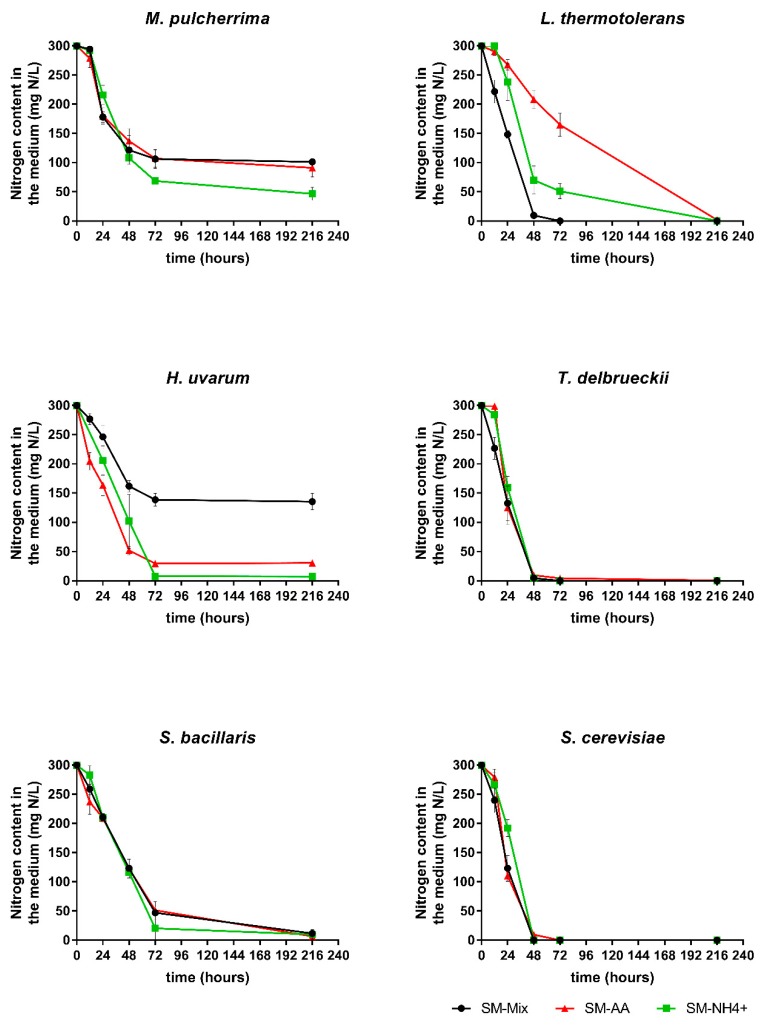
Nitrogen consumption (mg N/L) profiles of single fermentations in three different media: SM-Mix, SM-AA, and SM-NH_4_^+^. Error bars represent standard deviation.

Nitrogen consumption was measured throughout the different experimental fermentations (Figure 3). Sc, Td, and Sb showed similar consumption profiles, regardless of the nitrogen composition of the medium. Sc and Td depleted the nitrogen in 48 h, while Sb consumed most of the YAN during the first 72 h.

The other three species exhibited different consumption profiles of the YAN present in the media, depending on its composition. Lt depleted all YAN but showed different rates, with the fastest in SM-Mix and the slowest in SM-AA. On the other hand, none of the fermentations performed with Mp were able to consume all of the nitrogen, with residual nitrogen levels in the medium between 60 mg/L YAN (SM-NH_4_^+^) and 120 mg/L YAN (SM-Mix and SM-AA). Surprisingly, Hu consumed much less nitrogen in SM-Mix than in the other two media, although this difference was not reflected in the fermentation or growth kinetics (Figure 1).

**Figure 4:**
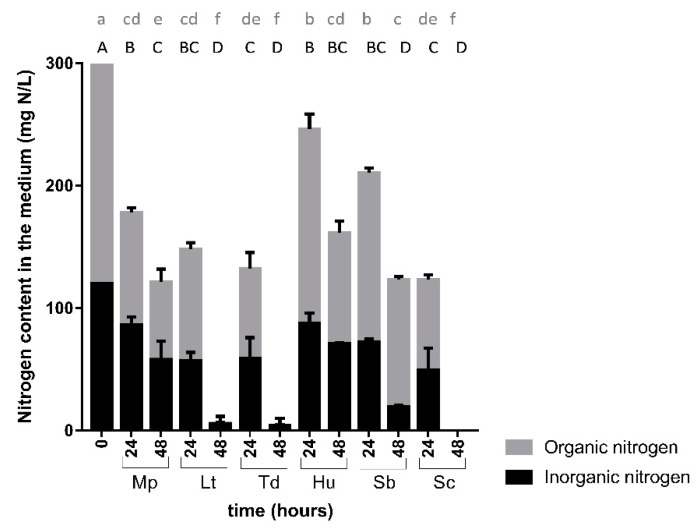
Organic and inorganic nitrogen (mg N/L) present in the medium at 0, 24, and 48 h during single fermentations in SM-Mix. Capital letters indicate significant differences in inorganic nitrogen levels. Lowercase letters indicate significant differences in organic nitrogen levels.

The preference between organic and inorganic nitrogen in SM-Mix differed among the species (Figure 4). Inorganic and organic nitrogen were similarly consumed by Sc, Td, and Lt, depleting all the nitrogen in less than 48 h. In contrast, the other three species (Mp, Hu, and Sb) consumed less than 200 mg N/L in the same period, with Hu being the strain with the lowest YAN uptake, and Sb the strain with the lowest amino acid consumption in 48 h.

**Figure 5:**
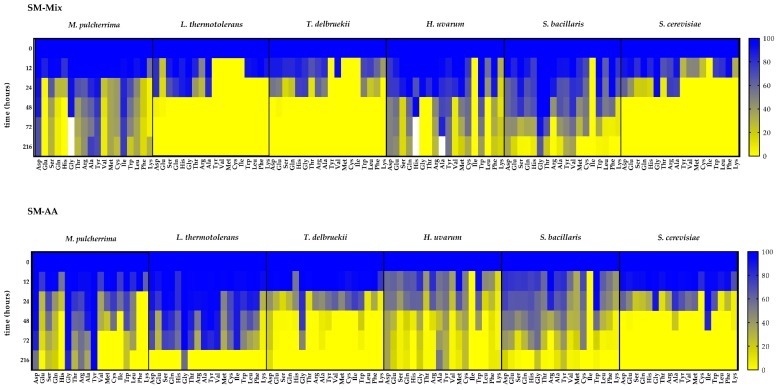
Amino acid concentration (%) present in the medium at different time points of the alcoholic fermentation in SM-Mix and SM-AA. The initial concentration of each amino acid is expressed as 100%. White color represents more than 100%.

To better understand the amino acid preferences of each species and how the presence of ammonium could modulate their uptake, we compared the amino acid consumption patterns of different species in the presence or absence of ammonium (Figure 5, Table S3 and S4).

**Figure 6:**
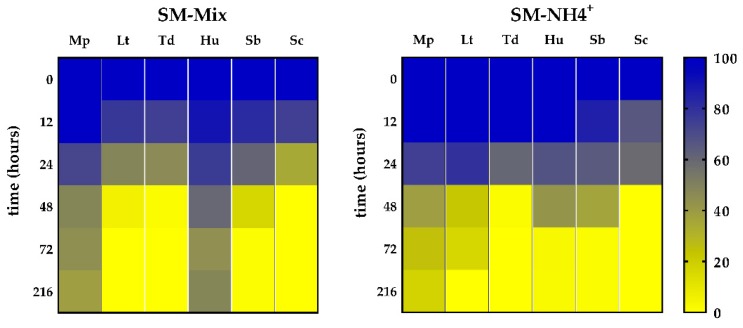
Ammonium concentration (%) present in the medium at different time points of the alcoholic fermentation in SM-Mix and SM-NH_4_^+^. The initial concentration of ammonium is expressed as 100%.

As expected, the amino acid consumption profile in SM-Mix differed from that in SM-AA. In SM-Mix, Td and Lt behaved similarly to Sc and were the fastest non-*Saccharomyces* species to consume all amino acids. Td and Lt first consumed tyrosine, methionine, cysteine, and isoleucine (in less than 12 h). Additionally, the consumption of aspartic acid, histidine, glycine, and alanine occurred mostly when ammonium was exhausted from the media (at 48 h, Figure 6). Surprisingly, in SM-AA, no amino acid uptake was observed during the first 12 h for Td and Lt (except for histidine). In this medium, Lt exhibited a strong delay in amino acid consumption, requiring more than 72 h for its depletion, except for glycine, which was not completely consumed under this condition. Indeed, the uptake of this amino acid was also delayed in Td, with its consumption beginning at 72 h.

As mentioned above, Sb was able to use all nitrogen in all media, while Hu fermentation had higher residual nitrogen levels in SM-Mix (135.36 ± 11.46 mg N/L) than in the other fermentations (Figure 3). Nevertheless, both species presented a similar amino acid consumption profile in both media, with depletion of isoleucine in less than 12 h, and a relatively fast amino acid uptake in the absence of ammonia (SM-AA). In fact, when ammonium was present, practically half of the organic nitrogen (mainly formed by alanine, glutamine, and arginine) remained in the medium in the fermentations with Hu, which even excreted some amino acids, such as alanine and histidine (Table S3). In the fermentations with Sb, we also observed a delay in the uptake of the amino acids, probably linked to the presence of ammonia. The least consumed amino acids by this species were glycine, tyrosine, aspartic acid, and valine.

Finally, the presence of ammonia in the media did not greatly affect the amino acid uptake in Mp. As observed in Sb, Mp was also not able to consume some amino acids, such as aspartic acid, tyrosine in any media, glycine in SM-AA, and alanine or isoleucine in SM-Mix.

Ammonium consumption was analyzed at the same time points as that of amino acids (Figure 6, Table S5). Sc and Td depleted ammonium in less than 48 h in both media. Lt and Sb fermentations consumed ammonium slowly in SM-NH_4_^+^. In contrast, Hu and Mp were not able to consume all ammonium when amino acids were present. Indeed, Mp was the only strain with residual ammonium present in SM-NH_4_^+^.

**Figure 7:**
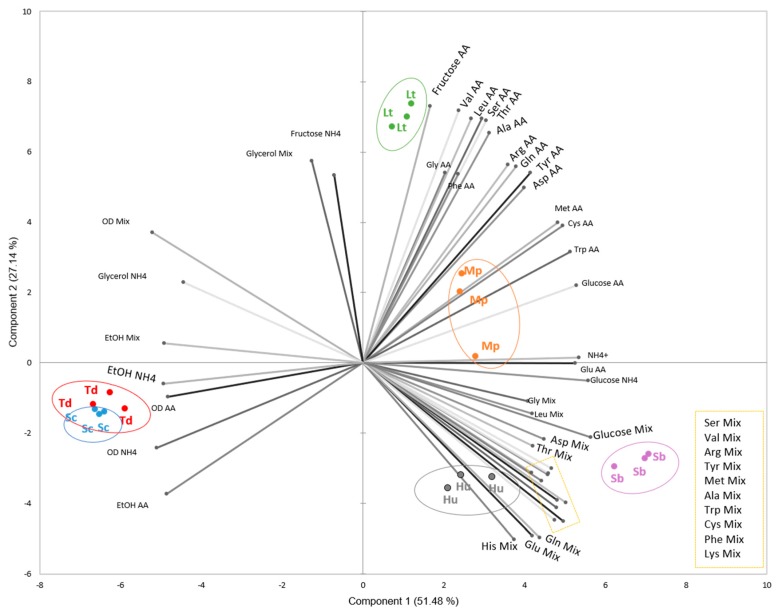
Biplot of principal component analysis (PCA) using nitrogen concentration, OD_600_, and the glucose, fructose, glycerol, and ethanol contents at 48 h in all media as variables. The explicative variables were distributed along the PCA axes as follows: Component 1 (+): glucose, aspartic acid (Asp), glutamic acid (Glu), serine (Ser), glutamine (Gln), histidine (His), glycine (Gly), threonine (Thr), arginine (Arg), alanine (Ala), tyrosine (Tyr), valine (Val), methionine (Met), cysteine (Cys), tryptophan (Trp), leucine (Leu), phenylalanine (Phe), lysine (Lys) (SM-Mix); glucose, Asp, Glu, Tyr, Met, Cys, Trp (SM-AA); glucose, ammonium (SM-NH_4_^+^). (−): OD_600_, ethanol (SM-Mix); OD_600_, ethanol (SM-AA), OD_600_, glycerol, ethanol (SM-NH_4_^+^). Component 2 (+): glycerol (SM-Mix); fructose, Ser, Gln, Gly, Thr, Arg, Ala, Val, Leu, Phe, (SM-AA); fructose (SM-NH_4_^+^).

For further analysis of these data, principal components analysis (PCA) was applied to correlate the different variables at 48 h and highlight if there were grouping patterns within the different species (Figure 7). The PCs explained 78.62% of the variance, and the variables that were positively and negatively correlated in each component are listed in Table S6. The six analyzed species clustered into five groups since Sc and Td grouped together and apart from the others due to their higher growth and ethanol contents and their lower glucose levels in all media, compared with those of other species (Component 1). On the other hand, Component 2 differentiated the other four species. The high levels of fructose in SM-AA and SM-NH_4_^+^, together with the low consumption of some amino acids in SM-AA, clearly separated Lt from the other species. However, when PCA was performed for each medium separately (Figure S2), Lt grouped with Sc and Td in the SM-Mix medium. Additionally, Hu and Sb were clustered on the negative axis of Component 2 due to their lower consumption of amino acids in SM-Mix than in the other media.

Finally, to compare the fermentative behavior of the different species in the three media used, some compounds of oenological interest were analyzed in the final wine (Table 2). In general, glucose was preferably consumed to fructose, although as expected, Sb showed a clear fructophilic behavior, and Hu seemed to consume fructose slightly faster than it consumed glucose. Sc and Td obtained the highest ethanol contents in all the media, although the ethanol yield was very similar in most species, except for Mp and Lt, which exhibited slightly lower yields in some media. In fact, Mp was the strain with the highest glycerol production in all media after 10 days of fermentation and was also the highest at 48 h (Table S7). SM-AA was the medium exhibiting the lowest glycerol production. On the other hand, the acetic acid content varied between 0.18 and 1.90 g/L, and Mp was also the strain that produced high acetic acid levels in all conditions, even though its lower sugar consumption, and Lt the one with the lowest production yield, in SM-Mix.

## 4. Discussion

Nitrogen utilization and metabolism have been extensively researched in *S. cerevisiae*. However, in the last decade, the use of non-*Saccharomyces* (NS) species has been widely spread in winemaking and has been used in mixed and/or sequential starter cultures due to their desirable properties, which could positively contribute to the quality of wine [46,47]. For this reason, knowledge of the utilization of nitrogen by NS species is critical for proper fermentation development in mixed or sequential cultures. Nevertheless, only a few and recent studies have evaluated the differential utilization of nitrogen by some NS yeasts [2,19,32,33,34], suggesting that nitrogen consumption and nitrogen preferences are dependent on the strain and fermentation conditions. Thus, in this study, we analyzed the nitrogen preferences of five NS strains belonging to yeast species of oenological interest (*M. pulcherrima*, *T. delbrueckii*, *L. thermotolerans*, *H. uvarum*, and *S. bacillaris*) in different nitrogen compositions of the media. We used strains that have not been previously analyzed, some of which are natural isolates from DOQ Priorat, to broaden the understanding of nitrogen metabolism of these species and improve the management of their implementation in wine fermentation.

*S. cerevisiae* was the fastest yeast to ferment all tested media, due to its well-known good fermentative capacity [13,48,49,50]. In SM-Mix, *T. delbrueckii* and *L. thermotolerans*, which also had good fermentation performance, exhibited a similar nitrogen consumption pattern to that of *S. cerevisiae*. These results were consistent with those of previous studies [12,32,33,51], where strong fermenter species, such as *T. delbrueckii*, *L. thermotolerans*, *K. marxianus*, or *S. paradoxus*, assimilated nitrogen in a similar manner as *S. cerevisiae*, while poorly fermenting species, such as *M. pulcherrima*, *P. kluyveri*, *P. burtonii*, or *Zygoascus meyerae*, displayed a low nitrogen uptake.

In *T. delbrueckii* and *L. thermotolerans*, the assimilation of some amino acids, known to be repressed by the nitrogen catabolism repression (NCR) system in *S. cerevisiae* (aspartic acid, alanine, arginine, and histidine), was also delayed and linked to ammonium depletion, suggesting a nitrogen regulation system similar to the one that is well described in *S. cerevisiae* [12,17,49,52]. For *S. cerevisiae*, the assimilation of preferred nitrogen sources can be explained mainly by the regulation of nitrogen transport, including the Ssy1p-Ptr3p-Ssy5 (SPS) system [53] and the nitrogen catabolism repression (NCR) system [43,54]. These mechanisms have been scarcely explored in NS wine yeasts, as only recent studies had been done in the *Hanseniaspora* genus [52,55]. In fact, Lleixa et al. [52] demonstrated that the NCR mechanism could also be present in some NS species, specifically in *H. vineae* [52]. However, Seixas et al. [55] evidenced that almost all *S. cerevisiae* amino acid-specific permeases were absent in *Hanseniaspora guilliermondii*, *Hanseniaspora opuntiae*, or *H. uvarum*, suggesting that *Hanseniaspora* species might favor the utilization of general nitrogen permeases instead of specific ones. Another recent study performed in *Kluyveromyces marxianus* [37] evidenced that the nitrogen regulation in this species was dissimilar to the one in *S. cerevisiae*, lacking some key ammonium permeases, such as Mep1 and Mep2. Thus, although the presence of an NCR system should not be ignored in those species, more studies would need to be performed.

On the other hand, the absence of ammonium in the medium resulted in a delay in the amino acids’ consumption for *T. delbrueckii*, *L. thermotolerans*, and *S. cerevisiae*, with higher differences among them, especially in *L. thermotolerans*, which could not finish the fermentation under this condition. These results do not agree with Prior et al. [33], who reported no difference in amino acids’ uptake in *L. thermotolerans* or *T. delbrueckii,* with and without ammonium. Under this condition, the latest amino acid consumed by *T. delbrueckii* and *L. thermotolerans* was glycine, which has been previously described as a poor nitrogen source for several yeast species [12,32,35,56,57]. Indeed, glycine was also one of the less preferred amino acids for *M. pulcherrima* and *S. bacillaris*, being even excreted/produced by *M. pulcherrima* in SM-Mix. In contrast, *H. uvarum* showed a preference for this amino acid, especially in SM-Mix. Kemsawasd et al. [32] also observed that glycine favored the consumption of glucose and the production of ethanol in *H. uvarum*, indicating that the preference for this amino acid is specific for this yeast species.

Ammonium is shown to be a good nitrogen source to support fermentation in *T. delbrueckii*, being consumed early in SM-Mix or SM-NH_4_^+^ [32,35], although yeast growth was impaired when fermenting with ammonia as a single nitrogen source. On the other hand, although *L. thermotolerans* also exhibited early assimilation of ammonia in mixed media, it was severely delayed in SM-NH_4_^+^. These results correlated with low yeast growth and fermentation kinetics in SM-NH_4_^+^, suggesting that this *Lachancea* strain might be favored by the presence of complete media containing organic and inorganic nitrogen. However, our fermentations performed with other *Lachancea* strains showed different growth and fermentation kinetics, indicating that the nitrogen preferences in *Lachancea* spp. seem to be strain-specific more than species-specific [19,32,51]. Some studies performed with other *Lachancea* strains confirmed this variability in this species, i.e., the *L. thermotolerans* strain BBMCZ7-FA20 completed the fermentation process slowly, in 21 days, in white must [51], and the *L. thermotolerans* Viniflora·ConcertoTM strain fermented well in musts with different nitrogen sources [33].

*H. uvarum*, *M. pulcherrima,* and *S. bacillaris* resulted in stuck fermentations under all conditions, which agrees with previous works performed with other strains of these species in single cultures [19,36,39]. Despite this, *S. bacillaris* was able to consume all nitrogen under all conditions. The nitrogen consumption profile of *S. bacillaris*, as well as that of *H. uvarum*, revealed a high delay in the assimilation of most amino acids in the presence of ammonium, which was not observed in *S. cerevisiae*, *T. delbrueckii*, or *L. thermotolerans*. Englezos et al. [36] described a similar behavior of two strains of *S. bacillaris*, suggesting differences in the regulation of nitrogen uptake between *S. bacillaris* and *S. cerevisiae*. Different explanations are proposed by these authors, such as less-efficient SPS-control methods of amino acid permeases, an inhibitory mechanism mediated by ammonium, or the use of an additional efficient system for ammonium uptake in *S. bacillaris* [36]. Indeed, the three species seemed to prefer a single inorganic nitrogen source, as they consumed more YAN in SM-NH_4_^+^ than in the other fermentation groups. Surprisingly, higher nitrogen consumption did not always correlate with higher growth or better fermentation kinetics. In fact, in our study, nitrogen uptake seemed to be decoupled from growth or fermentation under some conditions for *T. delbrueckii*, *H. uvarum,* or *S. bacillaris*. In particular, the growth of *S. bacillaris* was better sustained by amino acids than by ammonium, although similar consumption of YAN was observed. Those results indicated a less efficient conversion of inorganic nitrogen to growth by *S.bacillaris*. Gutierrez et al. [58] previously described a similar decoupled behavior between nitrogen uptake and growth in *S. cerevisiae* strains. These authors justified that part of the assimilated nitrogen was used by yeast to replenish intracellular pools rather than being utilized for reproduction. Therefore, our results demonstrated that, as observed in *S. cerevisiae*, nitrogen assimilation, yeast growth, and fermentation kinetics were not always correlated in non-*Saccharomyces* species, and it is important to study the effect of a nitrogen source on all these parameters.

The amino acid preferences of *M. pulcherrima* correlated with those outlined in previous studies performed with other strains [19,32,35], showing lysine, glutamine, valine, and glutamic acid as good nitrogen sources, and glycine, tyrosine, alanine, and aspartic acid as poor ones [35]. On the other hand, the consumption of isoleucine, which we and other authors observed as a good nitrogen source for most NS yeasts [19,32,35], seemed to be impaired in our *M. pulcherrima* strain in SM-Mix. This could be due to modifications of the matrix or fermentation conditions, as observed by Gobert et al. [19] with different temperatures, or to some genetic variations occurring among strains of the same species, as previously described for *S. cerevisiae* [59,60,61,62,63].

Interestingly, we also observed an increase of some amino acids after 72 h for *H. uvarum* (histidine, alanine, and lysine) and *M. pulcherrima* (glycine, alanine, and lysine), mainly in the presence of ammonia. However, we discarded this release of amino acids as a consequence of autolysis because it mainly occurred during the early stationary phase without an evident loss of viability. Previous studies have also shown a release of some amino acids in different NS species, such as *M. pulcherrima*, *T. delbrueckii*, and *Pichia kluyveri* [19], or *S. bacillaris* [36], although in some cases, this release was before uptake began, possibly due to a response to the stress of inoculation [64].

Overall, our results confirmed the high similarities between *S. cerevisiae* and *T. delbrueckii* under all conditions in terms of growth, fermentation profile, and nitrogen assimilation preferences. These similarities between *T. delbrueckii* and *S. cerevisiae* were not unexpected since these species are believed to have evolutionarily diverged approximately 100–150 million years ago [64], being very close genetically [65]. In fact, *T. delbrueckii* was previously included in *Saccharomyces* spp. as *Saccharomyces rosei* [66]. All these similarities could suggest that both species could share a similar nitrogen regulation system. Indeed, both species presented fast nitrogen assimilation, which could provide them with a competitive edge relative to other yeasts present in fermenting musts [67,68].

On the other hand, the *L. thermotolerans* strain was also very similar to *S. cerevisiae* and *T. delbrueckii* but only in the complete medium. When only organic or inorganic nitrogen was used, its behavior changed drastically, resulting even in stuck fermentation, highlighting the importance of fermentation conditions when characterizing NS strains for their potential use in winemaking.

*M. pulcherrima* was the NS strain least affected by the nitrogen composition of the medium, presenting a similar nitrogen assimilation profile, as well as fermentation kinetics in all tested media. Although this species could not finish the fermentations, it had the highest production of glycerol and acetic acid and the lowest ethanol yield. In fact, this species has been described as a Crabtree-negative yeast, with a preference for respiratory metabolism under aerobic conditions [10,69,70], and it is a good candidate for lowering the ethanol content in wines. This respiro-fermentative metabolism is linked to an increase in glycerol and acetic acid contents, which correlates with our results.

The other two poor fermentative strains, *H. uvarum* and *S. bacillaris*, shared a preference for fructose as carbon source, especially *S. bacillaris*, which has been previously described as a highly fructophilic yeast [36,71,72]. Moreover, both strains behaved similarly regarding amino acid uptake, which occurred earlier than that of the good fermentative species in the absence of ammonia. These results could imply a different nitrogen regulation system for these species, as already suggested by Englezos et al. [36], although more studies are needed to confirm this hypothesis.

These species would be used in winemaking as mixed or sequential inocula, as their fermentative capacity is, in general, limited. In sequential fermentations, *S. cerevisiae* is usually added after 24 or 48 h of NS inoculation. Thus, the knowledge of the nitrogen consumption by the NS strains during this period is crucial to avoid the fermentative problem. In this work, we differentiated two behaviors regarding nitrogen consumption. One group included the good fermentative strains, *T. delbrueckii*, *L. thermotolerans*, which consumed practically all nitrogen in 48 h (as *S. cerevisiae*). This high consumption of nitrogen by those species might compromise the success of a sequential fermentation with *S. cerevisiae*, as suggested by Rollero et al. [34], making the nitrogen supplementation an essential practice for the correct development of the fermentations. Indeed, when using good fermentative NS strains, the recommended inoculation time of *S. cerevisiae* would be around 24 h. The other group included the weaker fermentative strains, *M. pulcherrima*, *S. bacillaris,* and *H. uvarum*, which only consumed part of the nitrogen present in the must at 48 h, leaving more than 100 mg N/L in the media. This residual nitrogen could be enough for the proper growth of *S. cerevisiae*, making the nitrogen supplementation less necessary. In order to allow those weaker fermenters to have an impact on the final wines, the inoculation of *S. cerevisiae* should preferably be done after 48 h.

In conclusion, nitrogen consumption and preferences are species-dependent, and the nitrogen composition of the must has a direct effect on the fermentation profile and yeast growth. This work highlighted the importance of controlling nitrogen availability in sequential fermentations, as some nitrogen compounds might be depleted by non-*Saccharomyces* yeasts before *Saccharomyces* inoculation, impairing its growth and the performance of the fermentation. Thus, it is important to control the nitrogen availability in mixed fermentations before inoculating *Saccharomyces* to avoid problems, such as stuck or sluggish fermentations.

## Figures and Tables

**Table 1:** Maximum growth (expressed as OD_600_) during single fermentations under three nitrogen conditions (SM-Mix, SM-NH_4_^+^, and SM-AA), for each yeast species: *M. pulcherrima* (Mp), *L. thermotolerans* (Lt), *T. delbrueckii* (Td), *H*. *uvarum* (Hu), *S. bacillaris* (Sb), and *S. cerevisiae* (Sc).

	Mp	Lt	Td	Hu	Sb	Sc
SM-Mix	12.00 ± 2.27 ^A^	18.17 ± 1.96 ^A^	20.67 ± 0.56 ^A^	5.16 ± 0.51 ^B^	7.06 ± 0.67 ^A^	16.76 ± 0.70 ^A^
SM-NH_4_^+^	13.91 ± 1.03 ^A^	8.29 ± 3.27 ^B^	14.13 ± 0.60 ^B^	8.71 ± 0.71 ^A^	5.91 ± 1.45 ^A^	16.95 ± 1.05 ^A^
SM-AA	10.33 ± 2.54 ^A^	10.45 ± 2.15 ^B^	14.40 ± 2.87 ^B^	5.47 ± 1.12 ^B^	7.73 ± 0.59 ^A^	18.65 ± 2.03 ^A^

Capital letters indicate significant differences in each species between the three media. SM-Mix: a mixture of organic and inorganic nitrogen; SM-NH_4_^+^: inorganic nitrogen only; AM-AA: organic nitrogen only.

**Table 2:** Concentration of compounds of oenological interest at the end of each single fermentation under three nitrogen conditions (SM-Mix, SM-NH_4_^+^, and SM-AA) for each yeast species: *M. pulcherrima* (Mp), *L. thermotolerans* (Lt), *T. delbrueckii* (Td), *H. uvarum* (Hu), *S. bacillaris* (Sb), and *S. cerevisiae* (Sc).

		Glucose	Fructose	Glycerol	Glycerol Yield	Acetic Acid	Acetic Acid Yield	Ethanol	Ethanol Yield
		g/L	g/L	g/L	g/g	g/L	mg/g	% (v/v)	g/g
Mp	SM-Mix	31.81 ± 2.68	49.85 ± 2.88	10.30 ± 0.50	0.06 ± 0.04	1.90 ± 0.10	15.91 ± 0.99	5.36 ± 0.11	0.36 ± 0.01
	SM-AA	30.77 ± 3.24	51.76 ± 2.80	6.87 ± 0.76	0.05 ± 0.01	0.98 ± 0.16	7.61 ± 0.20	5.59 ± 0.70	0.32 ± 0.05
	SM-NH_4_^+^	25.35 ± 2.00	43.46 ± 2.69	8.32 ± 0.25	0.06 ± 0.00	0.86 ± 0.44	8.04 ± 2.66	6.03 ± 0.32	0.36 ± 0.01
Lt	SM-Mix	3.15 ± 0.12	2.12 ± 2.99	10.10 ± 0.40	0.03 ± 0.02	0.23 ± 0.20	1.20 ± 0.84	9.55 ± 1.34	0.39 ± 0.05
	SM-AA	22.14 ± 2.15	54.78 ± 5.22	4.25 ± 1.38	0.03 ± 0.01	1.01 ± 0.29	8.74 ± 0.21	6.3 ± 0.94	0.34 ± 0.06
	SM-NH_4_^+^	22.65 ± 0.16	50.08 ± 0.34	6.034 ± 1.12	0.04 ± 0	0.27 ± 0.08	1.58 ± 0.13	5.84 ± 0.48	0.32 ± 0.08
Td	SM-Mix	3.03 ± 0.11	0.52 ± 0.17	7.72 ± 0.59	0.04 ± 0	1.16 ± 0.14	5.88 ± 0.59	9.92 ± 0.76	0.40 ± 0.02
	SM-AA	3.10 ± 0.40	1.60 ± 2.20	4.81 ± 0.02	0.02 ± 0	1.32 ± 0.32	7.65 ± 0.31	10.44 ± 0.75	0.42 ± 0.03
	SM-NH_4_^+^	2.60 ± 0.28	3.10 ± 1.60	7.80 ± 1.11	0.04 ± 0	0.52 ± 0.21	3.31 ± 0.20	10.54 ± 0.29	0.43 ± 0.01
Hu	SM-Mix	38.78 ± 3.98	24.38 ± 2.29	5.70 ± 0.63	0.04 ± 0.01	0.18 ± 0.06	1.33 ± 0.40	6.79 ± 0.18	0.39 ± 0.02
	SM-AA	36.80 ± 1.00	28.92 ± 0.95	3.95 ± 0.60	0.03 ± 0	0.18 ± 0.03	1.29 ± 0.17	6.80 ± 0.46	0.37 ± 0.06
	SM-NH_4_^+^	32.98 ± 3.91	22.26 ± 1.99	6.31 ± 0.29	0.04 ± 0	0.30 ± 0.14	2.00 ± 0.99	7.47 ± 0.54	0.38 ± 0.03
Sb	SM-Mix	46.77 ± 6.72	1.24 ± 0.35	6.32 ± 1.21	0.04 ± 0.01	0.51 ± 0.26	3.60 ± 1.43	8.61 ± 1.32	0.41 ± 0.01
	SM-AA	17.60 ± 18.86	0.00 ± 0.00	6.08 ± 1.83	0.03 ± 0.01	0.39 ± 0.06	2.12 ± 0.16	9.09 ± 0.76	0.39 ± 0.02
	SM-NH_4_^+^	61.72 ± 12.05	0.00 ± 0.00	8.60 ± 0.97	0.06 ± 0.02	0.24 ± 0.20	2.65 ± 0.56	7.51 ± 0.83	0.43 ± 0.03
Sc	SM-Mix	3.17 ± 0.50	1.97 ± 1.90	7.00 ± 0.37	0.04 ± 0	0.66 ± 0.22	3.37 ± 0.95	9.73 ± 0.19	0.39 ± 0.01
	SM-AA	3.05 ± 0.09	2.10 ± 1.40	5.38 ± 0.45	0.03 ± 0	0.71 ± 0.28	3.64 ± 1.18	10.28 ± 0.19	0.42 ± 0.01
	SM-NH_4_^+^	3.61 ± 0.41	2.36 ± 1.74	7.98 ± 0.97	0.04 ± 0	0.82 ± 0.11	4.19 ± 0.44	9.85 ± 0.43	0.40 ± 0.01

## Supplementary Materials

The following are available online at https://www.mdpi.com/2076-2607/8/2/157/s1. Figure S1: Fermentation kinetics of different *Lachancea* strains in the three media, Figure S2: Biplot of principal components analysis using nitrogen concentration, OD_600_, glucose, fructose, glycerol, and ethanol at 48 h in each medium as variables, Table S1: Synthetic must composition used in the present work, Table S2: Ammonium content and amino acid stock solution content expressed as g/L and the corresponding nitrogen concentration in synthetic must in mg N/L, Table S3: Nitrogen presence (mgN/L) in SM-Mix medium during single fermentations, Table S4: Nitrogen presence (mgN/L) in SM-AA medium during single fermentations, Table S5: Ammonium presence (%) in SM-NH_4_^+^ and SM-Mix media during single fermentations, Table S6: Correlated variables of PCA, and Table S7: Compounds of oenological interest present at 48 h in SM-Mix, SM-AA, and SM-NH_4_^+^ media.

## References

[1] Ribéreau-Gayon P., Dubourdieu D., Donèche B., Lonvaud A. (2006). Handbook of Enology: The Microbiology of Wine and Vinifications.

[2] Lleixà J., Manzano M., Mas A., del Portillo M.C. (2016). *Saccharomyces* and non-*Saccharomyces* competition during microvinification under different sugar and nitrogen conditions. Front. Microbiol..

[3] Renault P., Coulon J., Moine V., Thibon C., Bely M. (2016). Enhanced 3-sulfanylhexan-1-ol production in sequential mixed fermentation with *Torulaspora delbrueckii/Saccharomyces cerevisiae* reveals a situation of synergistic interaction between two industrial strains. Front. Microbiol..

[4] Albertin W., Zimmer A., Miot-Sertier C., Bernard M., Coulon J., Moine V., Colonna-Ceccaldi B., Bely M., Marullo P., Masneuf-Pomarede I. (2017). Combined effect of the *Saccharomyces cerevisiae* lag phase and the non-*Saccharomyces* consortium to enhance wine fruitiness and complexity. Appl. Microbiol. Biotechnol..

[5] Andorrà I., Berradre M., Mas A., Esteve-Zarzoso B., Guillamón J.M. (2010). Effect of pure and mixed cultures of the main wine yeast species on grape must fermentations. Eur. Food Res. Technol..

[6] Lleixà J., Martín V., del Portillo M.C., Carrau F., Beltran G., Mas A. (2016). Comparison of Fermentation and Wines Produced by Inoculation of *Hanseniaspora vineae* and *Saccharomyces cerevisiae*. Front. Microbiol..

[7] Renault P., Coulon J., Revel G., De Barbe J., Bely M. (2015). Increase of fruity aroma during mixed *T. delbrueckii/S. cerevisiae* wine fermentation is linked to specific esters enhancement. Int. J. Food Microbiol..

[8] Gobbi M., Comitini F., Domizio P., Romani C., Lencioni L., Mannazzu I., Ciani M. (2013). *Lachancea thermotolerans* and *Saccharomyces cerevisiae* in simultaneous and sequential co-fermentation: A strategy to enhance acidity and improve the overall quality of wine. Food Microbiol..

[9] Contreras A., Hidalgo C., Henschke P.A., Chambers P.J., Curtin C., Varela C. (2014). Evaluation of non-*Saccharomyces* yeasts for the reduction of alcohol content in wine. Appl. Environ. Microbiol..

[10] Quirós M., Rojas V., Gonzalez R., Morales P. (2014). Selection of non-*Saccharomyces* yeast strains for reducing alcohol levels in wine by sugar respiration. Int. J. Food Microbiol..

[11] Medina K., Boido E., Dellacassa E., Carrau F. (2012). Growth of non-*Saccharomyces* yeasts affects nutrient availability for *Saccharomyces* cerevisiae during wine fermentation. Int. J. Food Microbiol..

[12] Rollero S., Bloem A., Ortiz-Julien A., Camarasa C., Divol B. (2018). Altered fermentation performances, growth, and metabolic footprints reveal competition for nutrients between yeast species inoculated in synthetic grape juice-like medium. Front. Microbiol..

[13] Bisson L.F. (1999). Stuck and sluggish fermentations. Am. J. Enol. Vitic..

[14] Carrau F.M., Medina K., Farina L., Boido E., Henschke P.A., Dellacassa E. (2008). Production of fermentation aroma compounds by *Saccharomyces cerevisiae* wine yeasts: Effects of yeast assimilable nitrogen on two model strains. FEMS Yeast Res..

[15] Fairbairn S., McKinnon A., Musarurwa H.T., Ferreira A.C., Bauer F.F. (2017). The Impact of Single Amino Acids on Growth and Volatile Aroma Production by *Saccharomyces cerevisiae* Strains. Front. Microbiol..

[16] González B., Vázquez J., Morcillo-Parra M.A., Mas A., Torija M.J., Beltran G. (2018). The production of aromatic alcohols in non-*Saccharomyces* wine yeast is modulated by nutrient availability. Food Microbiol..

[17] Jiranek V., Langridge P., Henschke P.A. (1995). Amino acid and ammonium utilization by *Saccharomyces cerevisiae* wine yeasts from a chemically defined medium. Am. J. Enol. Vitic..

[18] Bell S.J., Henschke P.A. (2005). Implications of nitrogen nutrition for grapes, fermentation and wine. Aust. J. Grape Wine Res..

[19] Gobert A., Tourdot-Maréchal R., Morge C., Sparrow C., Liu Y., Quintanilla-Casas B., Vichi S., Alexandre H. (2017). Non-*Saccharomyces* Yeasts nitrogen source preferences: Impact on sequential fermentation and wine volatile compounds profile. Front. Microbiol..

[20] Ingledew W.M., Kunkee R. (1985). Factors Influencing Sluggish Fermentations of Grape Juice. Am. J. Enol. Vitic..

[21] Butzke C.E., Bisson L.F. (2000). Diagnosis and rectification of stuck and sluggish fermentations. Am. J. Enol. Vitic..

[22] Martínez-Moreno R., Quirós M., Morales P., Gonzalez R. (2014). New insights into the advantages of ammonium as a winemaking nutrient. Int. J. Food Microbiol..

[23] Gutiérrez A., Chiva R., Sancho M., Beltran G., Arroyo-López F.N., Guillamón J.M. (2012). Nitrogen requirements of commercial wine yeast strains during fermentation of a synthetic grape must. Food Microbiol..

[24] Monteiro F., Bisson L. (1991). Biological assay of nitrogen content of grape juice and prediction of sluggish fermentations. Am. J. Enol. Vitic..

[25] Bell A.A., Ough C.S., Kliewer W.M. (1979). Effects on Must and Wine Composition, Rates of Fermentation, and Wine Quality of Nitrogen Fertilization of Vitis Vinifera Var. Thompson Seedless Grapevines. Am. J. Enol. Vitic..

[26] Torija M.J., Beltran G., Novo M., Poblet M., Rozès N., Mas A., Guillamón J.M. (2003). Effect of organic acids and nitrogen source on alcoholic fermentation: Study of their buffering capacity. J. Agric. Food Chem..

[27] Beltran G., Esteve-Zarzoso B., Rozès N., Mas A., Guillamón J.M. (2005). Influence of the timing of nitrogen additions during synthetic grape must fermentations on fermentation kinetics and nitrogen consumption. J. Agric. Food Chem..

[28] Mendes-Ferreira A., Mendes-Faia A., Leao C. (2004). Growth and fermentation patterns of *Saccharomyces cerevisiae* under different ammonium concentrations and its implications in winemaking industry. J. Appl. Microbiol..

[29] Vilanova M., Ugliano M., Varela C., Siebert T., Pretorius I.S., Henschke P.A. (2007). Assimilable nitrogen utilisation and production of volatile and non-volatile compounds in chemically defined medium by *Saccharomyces cerevisiae* wine yeasts. Appl. Microbiol. Biotechnol..

[30] Jiménez-Martí E., Aranda A., Mendes-Ferreira A., Mendes-Faia A., del Olmo M.L. (2007). The nature of the nitrogen source added to nitrogen depleted vinifications conducted by a *Saccharomyces cerevisiae* strain in synthetic must affects gene expression and the levels of several volatile compounds. Antonie Van Leeuwenhoek.

[31] Gobert A., Tourdot-Maréchal R., Sparrow C., Morge C., Alexandre H. (2019). Influence of nitrogen status in wine alcoholic fermentation. Food Microbiol..

[32] Kemsawasd V., Viana T., Ardö Y., Arneborg N. (2015). Influence of nitrogen sources on growth and fermentation performance of different wine yeast species during alcoholic fermentation. Appl. Microbiol. Biotechnol..

[33] Prior K.J., Bauer F.F., Divol B. (2019). The utilisation of nitrogenous compounds by commercial non-*Saccharomyces* yeasts associated with wine. Food Microbiol..

[34] Rollero S., Bloem A., Ortiz-Julien A., Camarasa C., Divol B. (2018). Fermentation performances and aroma production of non-conventional wine yeasts are influenced by nitrogen preferences. FEMS Yeast Res..

[35] Su Y., Seguinot P., Sanchez I., Ortiz-Julien A., Heras J.M., Querol A., Camarasa C., Guillamón J.M. (2020). Nitrogen sources preferences of non-*Saccharomyces* yeasts to sustain growth and fermentation under winemaking conditions. Food Microbiol..

[36] Englezos V., Cocolin L., Rantsiou K., Ortiz-Julien A., Bloem A., Dequin S., Camarasa C. (2018). Specific Phenotypic Traits of *Starmerella bacillaris* Related to Nitrogen Source Consumption and Central Carbon Metabolite Production during Wine Fermentation. Appl. Environ. Microbiol..

[37] Rollero S., Bloem A., Ortiz-Julien A., Bauer F.F., Camarasa C., Divol B. (2019). A comparison of the nitrogen metabolic networks of *Kluyveromyces marxianus* and *Saccharomyces cerevisiae*. Environ Microbiol..

[38] Seguinot P., Bloem A., Brial P., Meudec E., Ortiz-Julien A., Camarasa C. (2020). Analysing the impact of the nature of the nitrogen source on the formation of volatile compounds to unravel the aroma metabolism of two non-*Saccharomyces* strains. Int. J. Food Microbiol..

[39] Andorrà I., Berradre M., Mas A., Esteve-Zarzoso B., Guillamón J.M. (2012). Effect of mixed culture fermentations on yeast populations and aroma profile. LWT.

[40] Taillandier P., Lai Q.P., Julien-Ortiz A., Brandam C. (2014). Interactions between *Torulaspora delbrueckii* and *Saccharomyces cerevisiae* in wine fermentation: Influence of inoculation and nitrogen content. World J. Microbiol. Biotechnol..

[41] Alonso-del-Real J., Pérez-Torrado R., Querol A., Barrio E. (2019). Dominance of wine *Saccharomyces cerevisiae* strains over *S. kudriavzevii* in industrial fermentation competitions is related to an acceleration of nutrient uptake and utilization. Environ. Microbiol..

[42] Curiel J.A., Morales P., Gonzalez R., Tronchoni J. (2017). Different non-*Saccharomyces* yeast species stimulate nutrient consumption in *S. cerevisiae* mixed cultures. Front. Microbiol..

[43] Beltran G., Novo M., Rozes N., Mas A., Guillamon J. (2004). Nitrogen catabolite repression in during wine fermentations. FEMS Yeast Res..

[44] Gómez-Alonso S., Hermosín-Gutiérrez I., Esteban G.-R. (2007). Simultaneous HPLC Analysis of Biogenic Amines, Amino Acids, and Ammonium Ion as Aminoenone Derivatives in Wine and Beer Samples. J. Agric. Food Chem..

[45] Quirós M., Gonzalez-Ramos D., Tabera L., Gonzalez R. (2010). A new methodology to obtain wine yeast strains overproducing mannoproteins. Int. J. Food Microbiol..

[46] Ciani M., Comitini F., Mannazzu I., Domizio P. (2010). Controlled mixed culture fermentation: A new perspective on the use of non-*Saccharomyces* yeasts in winemaking. FEMS Yeast Res..

[47] Jolly N.P., Varela C., Pretorius I.S. (2014). Not your ordinary yeast: Non-*Saccharomyces* yeasts in wine production uncovered. FEMS Yeast Res..

[48] Bauer F.F., Pretorius I.S. (2000). Yeast Stress Response and Fermentation Efficiency: How to Survive the Making of Wine—A Review. S. Afr. J. Enol. Vitic..

[49] Beltran G., Torija M.J., Novo M., Ferrer N., Poblet M., Guillamón J.M., Rozès N., Mas A. (2002). Analysis of yeast populations during alcoholic fermentation: A six year follow-up study. Syst. Appl. Microbiol..

[50] Torija M.J., Rozès N., Poblet M., Guillamón J.M., Mas A. (2001). Yeast population dynamics in spontaneous fermentations: Comparison between two different wine-producing areas over a period of three years. Antonie Van Leeuwenhoek.

[51] De Koker S. (2015). Nitrogen Utilisation of Selected Non-*Saccharomyces* Yeasts and the Impact on Volatile Compound Production. Master’s Thesis.

[52] Lleixà J., Martín V., Giorello F., del Portillo M.C., Carrau F., Beltran G., Mas A. (2019). Analysis of the NCR Mechanisms in *Hanseniaspora vineae* and *Saccharomyces cerevisiae* During Winemaking. Front. Genet..

[53] Ljungdahl P.O. (2009). Amino-acid-induced signalling via the SPS-sensing pathway in yeast. Biochem. Soc. Trans..

[54] Marini A.-M., Soussi-Boudekou S., Vissers S., Andre B. (1997). A Family of Ammonium Transporters in *Saccharomyces cerevisiae*. Mol. Cell. Biol..

[55] Seixas I., Barbosa C., Mendes-Faia A., Güldener U., Tenreiro R., Mendes-Ferreira A., Mira N.P. (2019). Genome sequence of the non-conventional wine yeast *Hanseniaspora guilliermondii* UTAD222 unveils relevant traits of this species and of the *Hanseniaspora* genus in the context of wine fermentation. DNA Res..

[56] Cooper T.G., Strathern J.N., Jones E.W., Broach J.B. (1982). Nitrogen metabolism in *Saccharomyces cerevisiae*. The Molecular Biology of the Yeast Saccharomyces, Metabolism and Gene Expression.

[57] Ljungdahl P.O., Daignan-Fornier B. (2012). Regulation of amino acid, nucleotide, and phosphate metabolism in *Saccharomyces cerevisiae*. Genetics.

[58] Gutiérrez A., Sancho M., Beltran G., Guillamon J.M., Warringer J. (2016). Replenishment and mobilization of intracellular nitrogen pools decouples wine yeast nitrogen uptake from growth. Appl. Microbiol. Biotechnol..

[59] Cubillos F.A., Louis E.J., Liti G. (2009). Generation of a large set of genetically tractable haploid and diploid *Saccharomyces* strains. FEMS Yeast Res..

[60] Salinas F., Cubillos A., Soto D., Garcia V., Bergström A., Warringer J., Ganga M.A., Louis E.J., Liti G., Martinez C. (2012). The Genetic Basis of Natural Variation in Oenological Traits in *Saccharomyces cerevisiae*. PLoS ONE.

[61] Gutiérrez A., Beltran G., Warringer J., Guillamón J.M. (2013). Genetic Basis of Variations in Nitrogen Source Utilization in Four Wine Commercial Yeast Strains. PLoS ONE.

[62] Zimmer A., Durand C., Loira N., Durrens P., Sherman D.J., Marullo P. (2014). QTL dissection of lag phase in wine fermentation reveals a new translocation responsible for *Saccharomyces cerevisiae* adaptation to sulfite. PLoS ONE.

[63] Marullo P., Aigle M., Bely M., Masneuf-Pomarède I., Durrens P., Dubourdieu D., Yvert G. (2007). Single QTL mapping and nucleotide-level resolution of a physiologic trait in wine *Saccharomyces cerevisiae* strains. FEMS Yeast Res..

[64] Hagman A., Säll T., Piškur J. (2014). Analysis of the yeast short-term Crabtree effect and its origin. FEBS J..

[65] Masneuf-Pomarede I., Bely M., Marullo P., Albertin W. (2016). The Genetics of Non-conventional Wine Yeasts: Current Knowledge and Future Challenges. Front. Microbiol..

[66] Bely M., Stoeckle P., Masneuf-Pomarède I., Dubourdieu D. (2008). Impact of mixed *Torulaspora delbrueckii-Saccharomyces cerevisiae* culture on high-sugar fermentation. Int. J. Food Microbiol..

[67] Crépin L., Nidelet T., Sanchez I., Dequin S., Camarasa C. (2012). Sequential use of nitrogen compounds by *Saccharomyces cerevisiae* during wine fermentation: A model based on kinetic and regulation characteristics of nitrogen permeases. Appl. Environ. Microbiol..

[68] García-Ríos E., Gutiérrez A., Salvadó Z.Z., Arroyo-López F.N., Guillamon J.M. (2014). The Fitness Advantage of Commercial Wine Yeasts in Relation to the Nitrogen Concentration, Temperature, and Ethanol Content under Microvinification Conditions. Appl. Environ. Microbiol..

[69] Contreras A., Hidalgo C., Schmidt S., Henschke P.A., Curtin C., Varela C. (2015). The application of non-*Saccharomyces* yeast in fermentations with limited aeration as a strategy for the production of wine with reduced alcohol content. Int. J. Food Microbiol..

[70] Venturin C., Boze H., Moulin G., Galzy P. (1995). Influence of oxygen limitation on glucose metabolism in *Hanseniaspora uvarum* K5 grown in chemostat. Biotechnol. Lett..

[71] Englezos V., Rantsiou K., Cravero F., Torchio F., Ortiz-Julien A., Gerbi V., Rolle L., Cocolin L. (2016). Starmerella bacillaris and *Saccharomyces cerevisiae* mixed fermentations to reduce ethanol content in wine. Appl. Microbiol. Biotechnol..

[72] Mestre Furlani M.V., Maturano Y.P., Combina M., Mercado L.A., Toro M.E., Vazquez F. (2017). Selection of non-*Saccharomyces* yeasts to be used in grape musts with high alcoholic potential: A strategy to obtain wines with reduced ethanol content. FEMS Yeast Res..

