# Supplementary material for: Nitrogen Preferences during Alcoholic Fermentation of Different Non-Saccharomyces Yeasts of Oenological Interest
Source: Microorganisms. 2020 Jan 22;8(2):157. doi: 10.3390/microorganisms8020157 (PMC7074775; doi:10.3390/microorganisms8020157)
Supplement: Supplementary file 1 [file microorganisms-08-00157-s001.zip › Supplementary_Material/Tables S1 and S2.docx]

Supplementary Material

**Supplementary Table S1.** Synthetic must composition used in the present work.

|  |  | Concentration (g/L) |
| --- | --- | --- |
| Sugars | Glucose | 100 |
|  | Fructose | 100 |
| Acids | Citric acid | 0.5 |
|  | Malic acid | 5 |
|  | Tartaric acid | 3 |
| Salts | KH_2_PO_4_ | 0.750 |
|  | K_2_SO_4_ | 0.500 |
|  | MgSO_2_ 7 H_2_O | 0.250 |
|  | CaCl_2_ 2 H_2_O | 0.155 |
|  | NaCl | 0.200 |
| Oligo-elements | MnSO_4_ H_2_O | 0.004 |
|  | ZnSO_4_ 7 H_2_O | 0.004 |
|  | CuSO_4_. 5 _H2_O | 0.001 |
|  | KI | 0.001 |
|  | CoCl_2_ 6 H_2_O | 0.0004 |
|  | H_3_BO_3_ | 0.001 |
|  | (NH_4_)_6_Mo_7_O_24_ | 0.001 |
| Vitamins | Myo-inositol | 0.02 |
|  | Pantothenate calcium | 0.0015 |
|  | Thiamine hydrochloride | 0.00025 |
|  | Nicotinic acid | 0.002 |
|  | Pyridoxine | 0.00025 |
|  | Biotine | 0.000003 |
| Anaerobic factors | Ergosterol | 0.015 |
|  | Oleic acid | 0.0045 |
|  | Tween 80 | 0.535 |
|  | Ethanol (absolute) | 0.395 |
| Nitrogen (300 mg N/L) |  |  |
| SM-Mix |  |  |
|  | NH_4_Cl (120 mg N/L) | 0.46 |
|  | Amino acid solution (180 mg N/L) | 6.25 mL * |
| SM-AA |  |  |
|  | NH_4_Cl (0 mg N/L) | 0.00 |
|  | Amino acid solution (300 mg N/L) | 10.42 mL * |
| SM-NH4^+^ |  |  |
|  | NH_4_Cl (300 mg N/L) | 0.214 g |
|  | Amino acid solution (0 mg N/L) | 0.00 mL * |

* Volum added from Amino acids stock solution (Table S2).

**Supplementary Table S2.** Ammonium content and amino acid stock solution content expressed as g L^-1^ and the corresponding nitrogen concentration in synthetic must in mg N L^-1^.

| **Amino acid** | **g L^-1^*** | **mg N L^-1^** |
| --- | --- | --- |
| Asp | 4.42 | 2,91 |
| Glu | 11.96 | 7,12 |
| Ser | 7.80 | 6,50 |
| Gln | 49.92 | 59,84 |
| His | 3.38 | 1,91 |
| Gly | 1.82 | 2,12 |
| Thr | 7.54 | 5,54 |
| Arg | 36.79 | 55,50 |
| Ala | 14.56 | 14,31 |
| Tyr | 1.95 | 0.94 |
| Cis | 2.08 | 1.50 |
| Val | 4.42 | 3.31 |
| Met | 3.12 | 1.83 |
| Trp | 17.42 | 7.47 |
| Phe | 3.77 | 2.00 |
| Ile | 3.25 | 2.17 |
| Leu | 4.81 | 3.21 |
| Lys | 1.69 | 2.03 |
| Pro | 45.60 | 0,00 |
| **Total aas** |  | 180.22 |
| **Ammonia (NH_4_Cl)** | 0.46 | 120.00 |
| **Total N** |  | 300.82 |

**Supplementary Table S3.** Nitrogen presence (mgN/L) in SM-Mix medium during single fermentations.

*Excel document.*

**Supplementary Table S4.** Nitrogen presence (mgN/L) in SM-AA medium during single fermentations.

*Excel document.*

**Supplementary Table S5.** Ammonium presence (%) in SM-NH4+ medium during single fermentations.

*Excel document.*

**Supplementary Table S6.** Nitrogen presence (mgN/L) at 24h in SM-Mix and SM-AA mediums.

*Excel document*

**Supplementary Figure S1.** Fermentation kinetics of different *Lachancea* strains in the three mediums.

**Supplementary Figure S2**. Biplot of principal components analysis using nitrogen concentration, OD, Glucose, Fructose, Glicerol and Ethanol at 48 hours in each medium as variables.
