# Supplementary figures and images for: Nitrogen Preferences during Alcoholic Fermentation of Different Non-Saccharomyces Yeasts of Oenological Interest
Source: Microorganisms. 2020 Jan 22;8(2):157. doi: 10.3390/microorganisms8020157 (PMC7074775; doi:10.3390/microorganisms8020157)

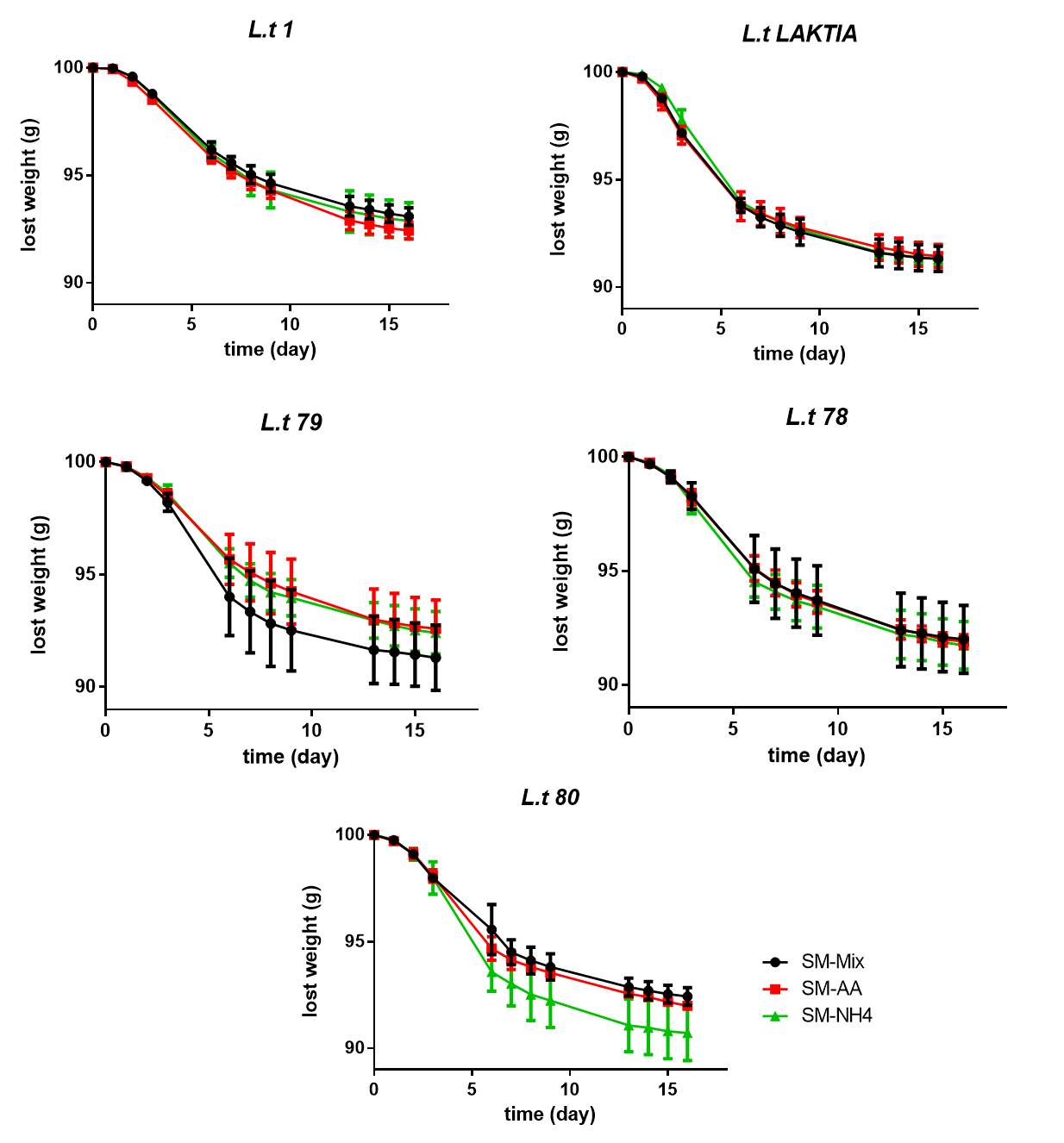

Supplement: Supplementary file 1 [file microorganisms-08-00157-s001.zip › Supplementary_Material/Figure S1.tif]

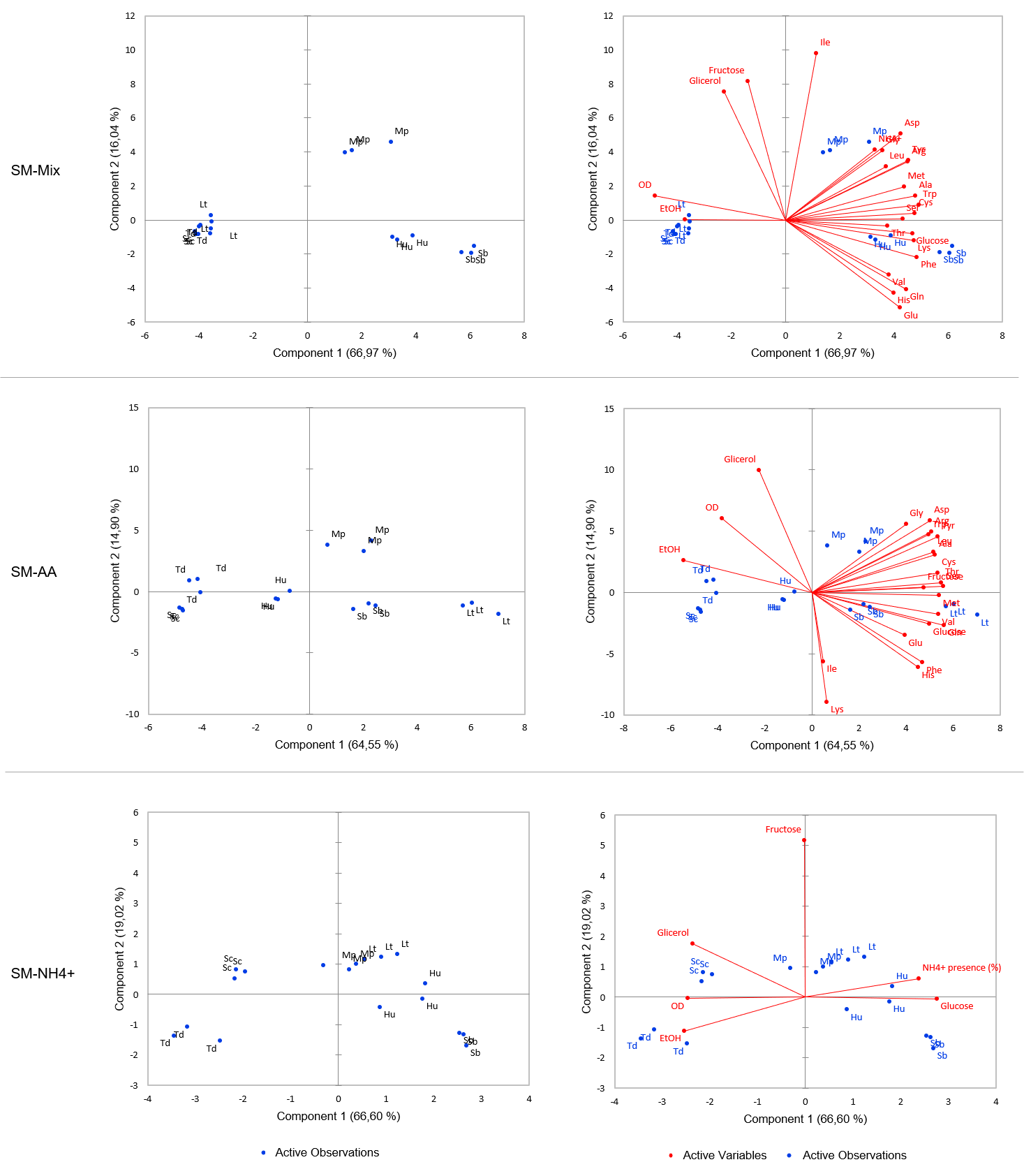

Supplement: Supplementary file 1 [file microorganisms-08-00157-s001.zip › Supplementary_Material/Figure S2.tif]
